# Patient-specific computational models of retinal prostheses

**DOI:** 10.1038/s41598-023-49580-6

**Published:** 2023-12-14

**Authors:** Kathleen E. Kish, Alex Yuan, James D. Weiland

**Affiliations:** 1 Biomedical Engineering, University of Michigan, Ann Arbor, 48105 USA; 2 BioInterfaces Institute, University of Michigan, Ann Arbor, 48105 USA; 3 Ophthalmology and Ophthalmic Research, Cole Eye Institute, Cleveland Clinic Foundation, Cleveland, 44195 USA; 4 Ophthalmology and Visual Science, University of Michigan, Ann Arbor, 48105 USA

**Keywords:** Biomedical engineering, Retina, Biophysical models

## Abstract

Retinal prostheses stimulate inner retinal neurons to create visual perception for blind patients. Implanted arrays have many small electrodes. Not all electrodes induce perception at the same stimulus amplitude, requiring clinicians to manually establish a visual perception threshold for each one. Phosphenes created by single-electrode stimuli can also vary in shape, size, and brightness. Computational models provide a tool to predict inter-electrode variability and automate device programming. In this study, we created statistical and patient-specific field-cable models to investigate inter-electrode variability across seven epiretinal prosthesis users. Our statistical analysis revealed that retinal thickness beneath the electrode correlated with perceptual threshold, with a significant fixed effect across participants. Electrode-retina distance and electrode impedance also correlated with perceptual threshold for some participants, but these effects varied by individual. We developed a novel method to construct patient-specific field-cable models from optical coherence tomography images. Predictions with these models significantly correlated with perceptual threshold for 80% of participants. Additionally, we demonstrated that patient-specific field-cable models could predict retinal activity and phosphene size. These computational models could be beneficial for determining optimal stimulation settings in silico, circumventing the trial-and-error testing of a large parameter space in clinic.

## Introduction

Electronic visual prostheses activate neurons in the visual pathway to create light perception for blind patients^1^. Electric current alters the transmembrane potential of nearby neurons, opening voltage-sensitive ion channels to induce action potentials. Artificially induced spots of light are called phosphenes, and visual prostheses aim to create interpretable scenes composed of phosphenes. Retinal prostheses are implanted at the back of the eye and activate inner retinal neurons for patients with degenerated photoreceptors^2^. Clinical testing has demonstrated that people with retinal prostheses can detect large objects, and in some cases distinguish letters, although at a rate much slower than natural reading^3^. Retinal prostheses still function ten years post-surgery, which supports the safety of long-term implantation and stimulation^4^.

Despite advancements in the field of artificial vision, retinal prostheses continue to face substantial limitations. Even the best-restored visual acuity is below the threshold of legal blindness^5^. The future success of these devices depends on their ability to activate target neurons with improved spatiotemporal resolution, while avoiding off-target stimulation^6^. To achieve this, researchers are developing microelectrode arrays with an increasing number of electrical contacts. Early commercial retinal prostheses have fewer than one hundred electrodes^7^. However, future generation implants could have hundreds or thousands^2^. Not all electrodes will induce a percept at the same current amplitude, requiring the establishment of a visual perception threshold for each individual electrode. Manually programming these devices in clinic using a trial-and-error process will place an excessive burden on both clinicians and patients. Furthermore, phosphenes created by single-electrode stimuli can vary in shape, size, and brightness^8^.

Computational models provide a tool to predict inter-electrode variability and automate device programming. Prior statistical models have consistently found that perceptual threshold increases with electrode-retina distance^9–14^. Prior models have also used impedance and retinal thickness to predict perceptual thresholds with contradictory results^9–14^. Rizzo et al. found a fibrosis-like hyper reflective tissue at the array interface on about half of implanted devices, but no studies have investigated the effect of this fibrotic tissue on perceptual thresholds^15^. Other data-driven models have been used to predict the visual perception resulting from a set of retinal stimulation parameters^16,17^ or from a longitudinal dataset with dozens of clinical features^18^.

An alternative to the data-driven approach, field-cable models aim to explicitly model the retinal circuitry and its response to stimulation^19–25^. This two-part technique uses a three-dimensional bioelectric field model to solve for the spatial distribution of electric potential throughout the tissue, before calculating the effects on neural activity with cellular cable models^26^.

For this project, we analyzed data from seven subjects implanted with the Argus II retinal prosthesis (Second Sight Medical Products Inc., Sylmar, CA). Retinal anatomy and array position in our subject group were consistent with previous documentation of 33 Argus II users across 16 surgical sites^27^. While this device is no longer manufactured, hundreds of patients implanted over the past two decades provide a valuable resource for understanding epiretinal stimulation. The Argus II is a 60-channel epiretinal implant consisting of 200 µm platinum electrodes with a 525 µm pitch^28^. In this study, we investigated inter-electrode variability across subjects with both statistical and patient-specific field-cable models. Informed by prior work, we chose the following explanatory variables for our statistical model: electrode impedance, electrode-retina distance, retinal thickness beneath the electrode, and fibrotic tissue thickness. We constructed field-cable models as described in our prior work^29^. We evaluated the utility of these two computational approaches for predicting perceptual threshold. Additionally, we demonstrated that patient-specific field-cable models constructed from imaging data are useful for predicting retinal activity and phosphene size.

## Results

### Statistical models for perceptual threshold

**Figure 1:**
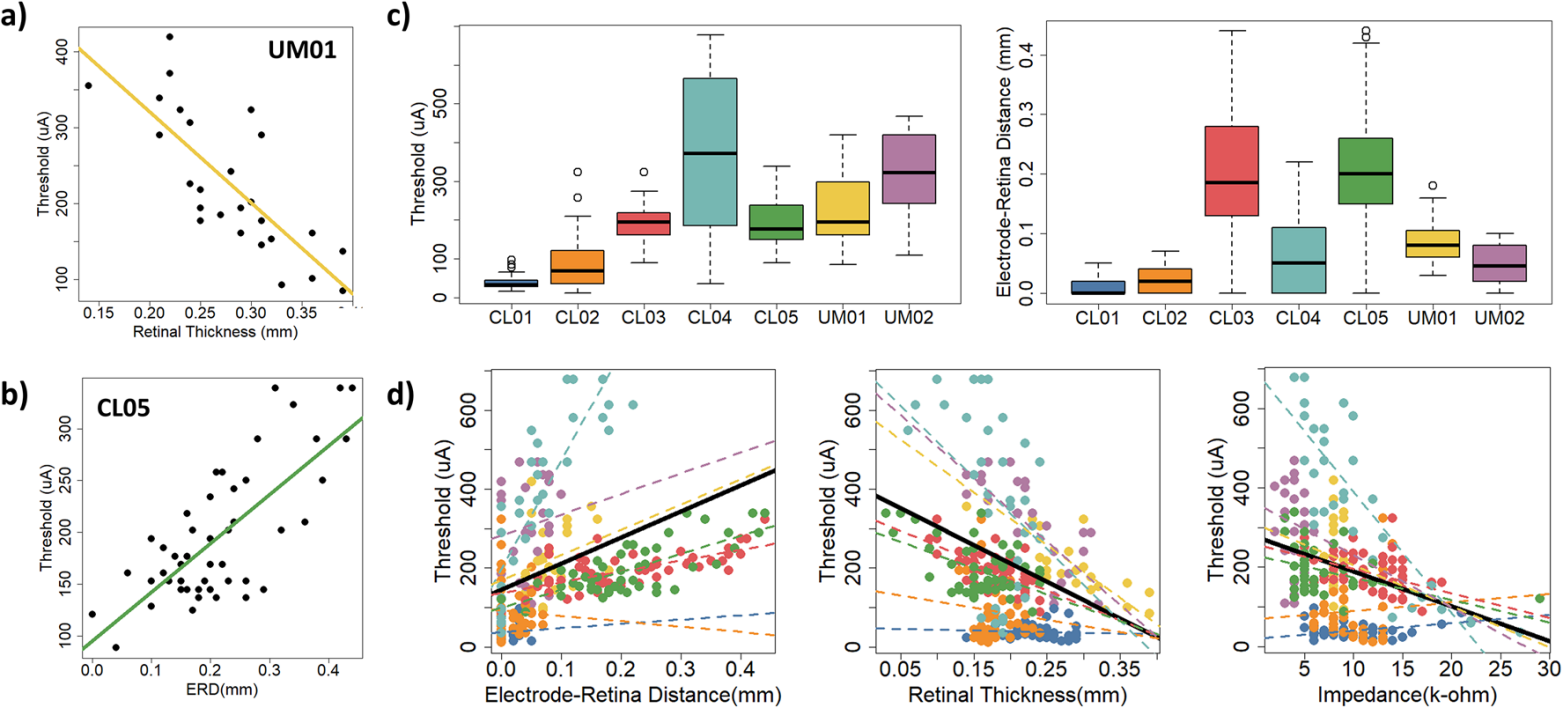
Statistical models of perceptual threshold. (**a**) Threshold versus retinal thickness for UM01. (**b**) Threshold versus electrode-retina distance for CL05. (**c**) Distribution of perceptual threshold and electrode-retina distance across participants. (**d**) Linear mixed models for electrode-retina distance, retinal thickness, and impedance. Data points are color-coded by participant, with dashed lines to show individual trends. Overall trend lines are shown in black.

We created linear regression models to determine the influence of four explanatory variables on visual perception threshold for epiretinal disc electrodes. The explanatory variables were electrode impedance, electrode-retina distance, retinal thickness beneath the electrode, and fibrotic tissue thickness on the electrode surface (if present). We collected data from seven participants implanted with the Argus II retinal prosthesis (Second Sight Medical Products Inc., Sylmar, CA). Table 1 summarizes the results of our regression analyses, including coefficients of determination and p-values. For most participants (5/7), there was a significant negative correlation between retinal thickness and perceptual threshold (i.e., Fig. 1a). For some participants (3/7), there was a significant positive correlation between electrode-retina distance and perceptual threshold (i.e., Fig. 1b). Two participants demonstrated a significant negative correlation between electrode impedance and perceptual threshold. Counter to our expectation, we found no significant correlation between fibrotic tissue thickness and perceptual threshold.

**Table 1:** Summary of coefficients of determination (R^2^) and *p*-values for regression analysis.

Participant ID	n	Electrode-retina distance	Retinal thickness	Electrode impedance	Fibrotic tissue thickness	Multiple regression
UM01	27	R^2^ = 0.06	R^2^ = 0.60*	R^2^ = 0.00	R^2^ = 0.11	R^2^ = 0.63*
		*p* = 0.24	*p* < 0.0001	*p* = 0.76	*p* = 0.09	*p* = 1.4e-4
UM02	26	R^2^ = 0.00	R^2^ = 0.52*	R^2^ = 0.02	R^2^ = 0.00	R^2^ = 0.62*
		*p* = 0.84	*p* < 0.0001	*p* = 0.49	*p* = 0.79	*p* = 3.1e-4
CL01	56	R^2^ = 0.01	R^2^ = 0.00	R^2^ = 0.00	R^2^ = 0.04	R^2^ = 0.11
		*p* = 0.45	*p* = 0.99	*p* = 0.99	*p* = 0.12	*p* = 0.12
CL02	45	R^2^ = 0.01	R^2^ = 0.04	R^2^ = 0.02	R^2^ = 0.02	R^2^ = 0.05
		*p* = 0.61	*p* = 0.19	*p* = 0.35	*p* = 0.44	*p* = 0.71
CL03	54	R^2^ = 0.45*	R^2^ = 0.43*	R^2^ = 0.14*	n/a^†^	R^2^ = 0.55*
		*p* < 0.0001	*p* < 0.0001	*p* = 5.6e-3		*p* < 0.0001
CL04	35	R^2^ = 0.71*	R^2^ = 0.23*	R^2^ = 0.72*	n/a^†^	R^2^ = 0.85*
		*p* < 0.0001	*p* = 3.4e-3	*p* < 0.0001		*p* < 0.0001
CL05	47	R^2^ = 0.55*	R^2^ = 0.23*	R^2^ = 0.08	n/a^†^	R^2^ = 0.57*
		*p* < 0.0001	*p* = 6.1e-4	*p* = 0.06		*p* < 0.0001
***Linear Mixed Model***	290	R^2^ = 0.12	R^2^ = 0.10	R^2^ = 0.05		
		Fixed Effect:	Fixed Effect:	Fixed Effect:		
		658.7 ± 409.9	− 929.6 ± 284.7*	− 8.8 ± 4.5		
		*p* = 0.16	*p* = 0.02	*p* = * p* = 0.09		

^†^Participant did not have fibrotic tissue growth on the array surface.

Based on the results of our multiple regression analysis, the measured explanatory variables accounted for 55–85% of the variability in perceptual threshold for all but two participants. For CL01 and CL02, they only explained 5–11% of the variability in perceptual threshold. It is worth noting the restricted range of electrode-retina distances and perceptual thresholds for these two participants (Fig. 1c). In other words, the epiretinal electrodes were close to the retina and perceptual thresholds were generally low. We also did not have access to clinical data (e.g., patient history, age, time since implant, etc.) that may affect perceptual threshold^18^.

We fit linear mixed models with random intercept and slope to determine if the influence of explanatory variables on threshold was non-zero on average, and if the effect varied across participants (Fig. 1d). Only retinal thickness had a significant fixed effect on perceptual threshold (*p* = 0.02). The effects on perceptual threshold of electrode-retina distance and electrode impedance varied by individual (*p* < 0.0001).

### Patient-specific field-cable models to predict perceptual threshold

From the same group of subjects, we built patient-specific field-cable models for five eyes implanted with the Argus II retinal prosthesis^24^. We excluded two subjects because no significant explanatory variables were identified during regression analysis. We constructed three-dimensional models from imaging data and used a two-part technique to model the electrical stimulation of retinal tissue. First, we calculated the electric fields generated by each stimulating electrode using finite element analysis^24,26,30,31^. Second, we functionalized the bulk tissue models with multi-compartment cable models of retinal ganglion cells (RGCs) to predict neural activity in response to the electric fields^24^. Using these models, we calculated the action potential threshold for RGCs beneath each stimulating electrode.

**Figure 2:**
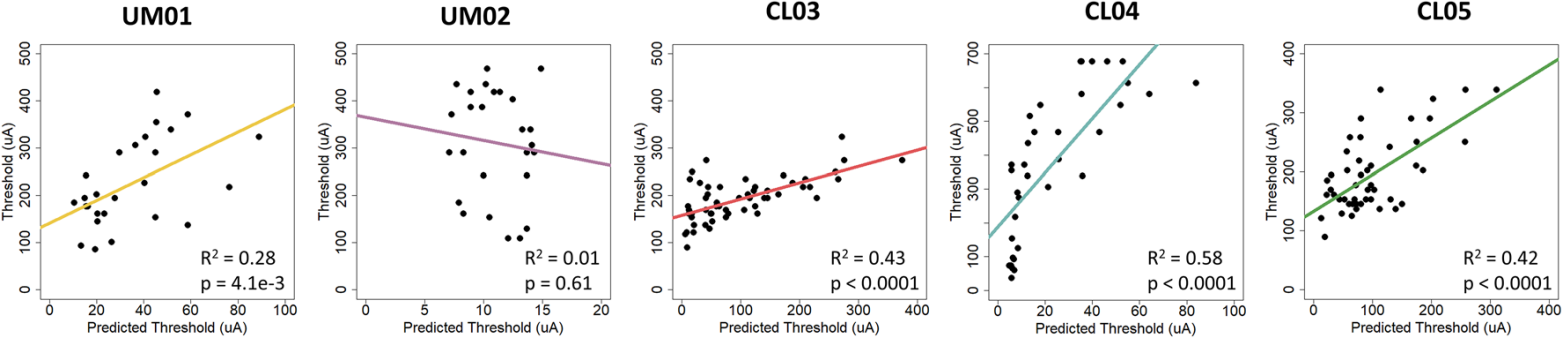
Linear regression analysis comparing visual perception thresholds with action potential thresholds in the patient-specific field-cable models.

We compared the action potential thresholds predicted by patient-specific field-cable models with participant visual perception thresholds using linear regression (Fig. 2). Our model predictions were significantly correlated with perceptual data for 4/5 participants.

In general, thresholds predicted in silico were lower than human reported thresholds. This was especially true when the electrode-retina distance was less than 100 µm. Changes to model conductivity values within the biological range did not eliminate this discrepancy^32,33^. For example, doubling (σ_retina_ = 0.2 S/m) and reducing (σ_retina_ = 0.01 S/m) the retinal conductivity caused only a 20.6% increase and 40.7% decrease in average predicted threshold. This may partially explain why the model did not correlate with human perceptual data for participant UM02. The average electrode-retina distance was 50 µm, and the model predicted neural activation in the range of 5–15 µA. However, the perceptual thresholds reported by the participant ranged from 100 to 500 µA. The model cannot explain these high thresholds, and may not be accounting for other factors that influence perceptual threshold (e.g., cortical magnification and the subjectivity of patient reporting). On the other hand, the electrode-retina distances for participant CL03 varied between 10 and 440 µm, and the model predicted neural activation in the range of 5–375 µA. This is closer to the range of perceptual thresholds reported by the participant (90–323 µA).

### Patient-specific field-cable models to predict phosphene size

**Figure 3:**
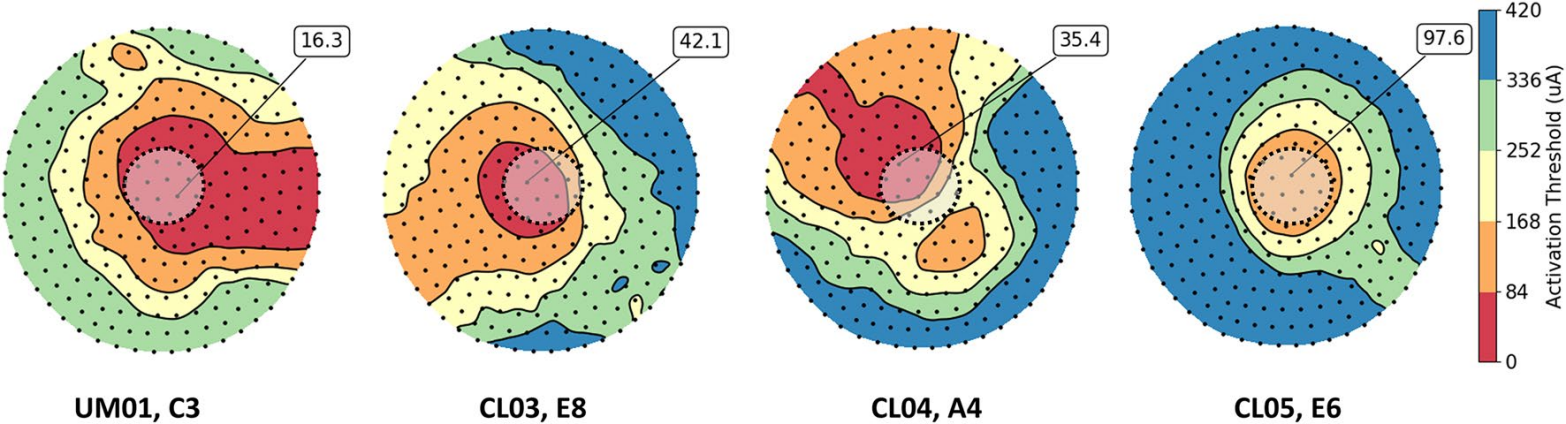
Thresholds predicted by the patient-specific field-cable models for four electrodes (dashed black line). Retinal ganglion cell somas are shown as black dots, and colored contours show the distribution of action potential thresholds beneath each electrode. The absolute minimum action potential threshold for each electrode is noted.

In addition to threshold prediction, the patient-specific field-cable models can estimate the activation patterns of retinal tissue (Fig. 3). These patterns depend on the position of the electrode with respect to the retina and the angle of nearby retinal ganglion cell axons^16^.

**Figure 4:**
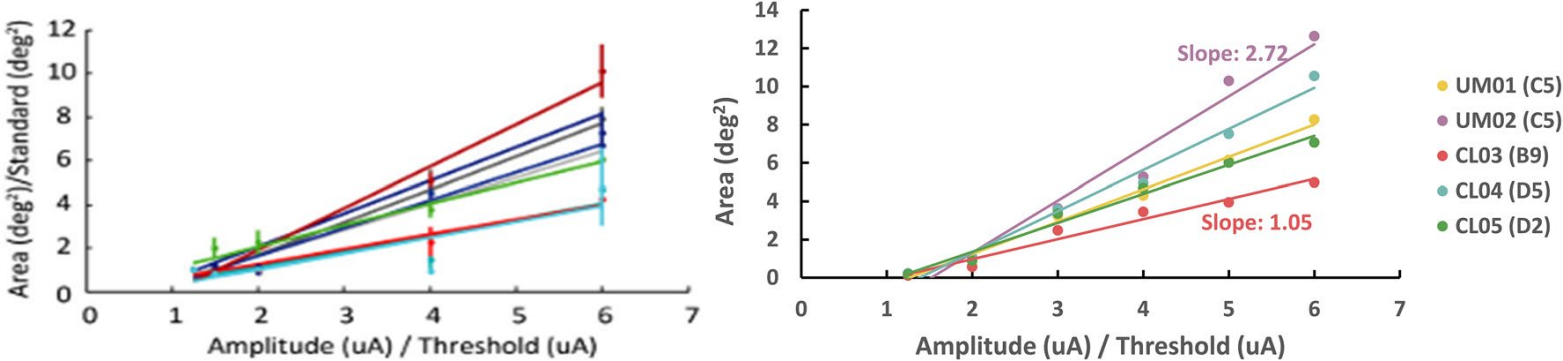
The effect of increasing stimulus amplitude on phosphene size. (**a**) Results from Nanduri et al.^8^ showing a linear increase of participant phosphene drawing size with stimulus amplitude. (**b**) Results from our patient-specific field-cable models, showing a linear increase of retinal area activated with stimulus amplitude, with slope varying across electrodes.

We compared the patient-specific field-cable model predictions of retinal activation to a prior study analyzing the effect of increasing stimulation amplitude on phosphene size^8^. The prior study found that increasing pulse amplitude caused an increase in phosphene size at an average rate of 1.17 deg^2^/X threshold, with the slope varying slightly across electrodes (Fig. 4a). The study used data from nine electrodes in a single Argus I patient^8^. Our models predicted a similar pattern of amplitude modulating phosphene size with slope varying between 1.05 and 2.72 deg^2^/X threshold across participants and electrodes (Fig. 4b).

## Discussion

Retinal prosthesis users report a variety of visual perception thresholds and phosphene shapes^34,35^. In this work, we developed statistical and patient-specific field-cable models to predict inter-patient and inter-electrode variability. Such models, used separately or in combination, have the potential to expedite the process of “fitting” a prosthesis for each patient, taking into account their particular anatomy and implant position.

Statistical models are a data-driven approach for predicting perceptual threshold. De Balthasar et al. previously investigated the effect of electrode-retina distance, retinal thickness, and impedance on perceptual threshold for six Argus I users^36^. The authors found a significant correlation between electrode-retina distance and threshold for 1/6 participants, between retinal thickness and threshold for 3/6 participants, and between impedance and threshold for 5/6 participants^36^. Other prior studies have confirmed the correlation between electrode-retina distance and perceptual threshold but have measured distance using the entire electrode array^35^ or groups of four electrodes^37^. In this study, we examined the correlation of electrode-retina distance, retinal thickness, fibrotic tissue thickness, and electrode impedance with perceptual threshold for individual epiretinal disc electrodes using regression analysis. One novel finding was that perceptual threshold increases systematically as retinal thickness decreases, with a fixed effect across participants. This suggests that retinal thickness could be a proxy for retinal health, and the number of viable neurons in some cases. Therefore, we recommend pre-operative OCT to guide array placement toward healthy retinal regions. Our study does not take into account the effects of cystoid macular edema, which could dynamically change the thickness measurements and could potentially explain some of the variability seen between patients. In addition, our regression analysis corroborates prior studies that identified an effect of electrode-retina distance on perceptual threshold, but we found that the strength and slope of correlation varies significantly across individuals. Finally, we found that fibrotic tissue growth on the surface of the microelectrode array did not affect perceptual threshold. This supports the findings of Rizzo et al., who found that 50% of Argus II users developed a fibrosis-like hyper reflective tissue at the array interface, but that those patients did not experience any deterioration in visual performance^38^. The effect of glial scarring on the performance of neural interfaces is a subject of debate. While increased impedance of chronically implanted electrodes can be associated with gliosis, it is unclear if gliosis is the direct cause of impedance increase^39^. A computational study conducted by Malaga et al. concluded that gliosis was not a main contributor toward increasing electrode impedance, and therefore should not cause an electrical problem with regard to signal quality^40^. These results further support our findings.

In general, data-driven models take an experimental dataset and fit a filter to capture the relationship between input and output variables. This approach is effective because it can be quick and does not require prior knowledge of the system. However, data-driven models are essentially a black box, providing limited understanding of the underlying physiological mechanisms.

On the other hand, pairing three-dimensional bioelectric field models with multi-compartment cable models aims to predict thresholds and phosphene patterns using first principles of electrophysiology. These predictions emerge directly from imaging data in absence of any perceptual data. The models can be used to efficiently map thresholds for all electrodes on an implanted array. Our patient-specific field-cable models predicted activation thresholds that significantly correlated with perceptual thresholds for 4/5 participants. However, our results show that the slope of the correlation is not consistent across participants (Fig. 2). There are other cases in the field of neuromodulation where each patient-specific field-cable model was fit with its own optimal conductivity value to improve performance^41^. This indicates that some perceptual data collection may be required to calibrate the models, even with the patient-specific field-cable approach that could conceivably produce threshold predictions based solely on imaging data. There was also a difference between the minimum thresholds predicted by our model (5 µA) and the lowest patient-reported thresholds (90 µA). This discrepancy can have several explanations. Individual device users have unique perceptual criteria for reporting phosphenes, and we do not know how many neurons must fire to produce a phosphene. Furthermore, RGC multi-compartment cable models have been developed using cell physiology data and are known to predict thresholds that are orders of magnitude lower than clinical research studies^42–44^. In summary, our patient-specific field-cable models did not predict threshold better than the statistical models; the prediction accuracy was similar. However, they provided direct estimates of retinal activity that relates to the shape and size of phosphenes.

The impact of this work includes expanding our novel proof-of-concept study conducted in a single participant to a cohort of five participants^24^ while also refining the original approach for greater efficiency. Our original work used both ultrasound and optical coherence tomography imaging to create a patient-specific field-cable model. We later found that ultrasound images, which we used to locate electrical ground, did not affect model predictions. Another improvement to our prior study was implementing a mammalian RGC model with dendrites that we optimized for run-time based on our sensitivity analysis, as opposed to a simplified, amphibian RGC model^45^.

Future work could apply this modelling framework to other retinal prostheses, for any device with OCT images available. An opaque epiretinal device might be more difficult to image. In addition, we could apply optimization paradigms to these models to program patient-specific stimulation settings. For example, we could evaluate multi-electrode paradigms that attempt to focalize phosphenes^6^. This virtual device programming session would help avoid manually testing a large parameter space in the clinic. Our results could also be used to inform simulations of prosthetic vision and reproduce inter-patient differences in the simulated visual scene^46^.

This study had several limitations. Current optical coherence tomography images cannot provide cellular-scale imaging of the retina, and therefore cannot directly measure the number of viable RGCs^47^. Although we identified a significant, fixed effect of retinal thickness on perceptual thresholds, we had to estimate the relationship between retinal thickness and neuron density. Removing RGCs from the model where the retina was less than 100 µm produced a good match with experimental data. However, the development of high-resolution retinal imaging that could directly measure cell count would improve our modelling framework. Secondly, our model did not include other inner retinal neurons (i.e., bipolar, amacrine, and horizontal cells). We assumed that epiretinal prostheses primarily cause direct RGC activation^1^. However, if this modelling framework were applied to intraretinal or subretinal prostheses, we should include a degenerate retinal network model^48,49^. A limitation of the patient-specific field-cable modelling approach is the time needed to build and run these models. To use them at a large-scale, we would recommend further automating our methodology.

## Methods

### Data collection

For this study, we obtained data from seven subjects implanted with the Argus II retinal prosthesis (Second Sight Medical Products Inc., Sylmar, CA). We recruited two subjects from the W.K. Kellogg Eye Center (University of Michigan, Ann Arbor, MI) and collected data for the purpose of this study (Clinical Trial ID: NCT03635645). We obtained informed consent following approval from the University of Michigan’s Institutional Review Board. The study adhered to the tenets of the Declaration of Helsinki and national regulations for medical device clinical trials. Five subjects were patients at the Cole Eye Institute (Cleveland Clinic, Cleveland, OH). Previously collected de-identified data was extracted from medical records, as specified by a data transfer and use agreement.

We obtained optical coherence tomography (OCT) scans of the implanted eye for each study participant. For UM01 and UM02, scans were taken using a Heidelberg Engineering Spectralis system. Images spanned 30° × 25° of the visual field, using 62 B-scans. Each B-scan was 768 pixels (8.8 mm) by 496 pixels (1.9 mm) and the scan-to-scan spacing was 122 µm. For the remaining participants, scans were taken using a Zeiss Cirrus system. Images spanned 20° × 20° of the visual field, using 128 B-scans. Each B-scan was 400 pixels (6 mm) by 120 pixels (2 mm) and the scan-to-scan spacing was 46.875 µm.

**Figure 5:**
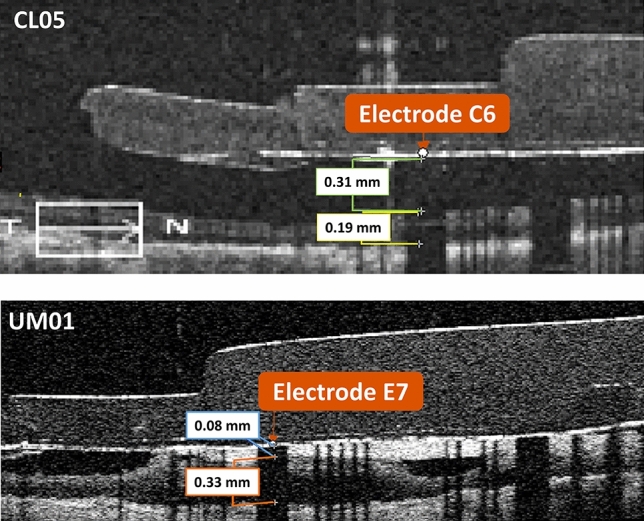
Optical coherence tomography scans with measurements. The top image shows a B-scan for participant CL05, with measurements for electrode-retina distance (0.31 mm) and overall retinal thickness (0.19 mm) for electrode C6. The bottom image shows a B-scan for participant UM01, with measurements for fibrotic tissue thickness (0.08 mm) and retinal thickness (0.33 mm) for electrode E7. In this case, the microelectrode array is opposed to the retina and the electrode-retina distance is equal to the fibrotic tissue thickness.

First, we used the OCT scans to make measurements of electrode-retina distance, retinal thickness, and fibrotic tissue thickness for all visible electrodes. The Argus II implant has sixty electrodes arranged in a 6 × 10 grid, with the standard labels A1-F10 (see Fig. 8a). We made measurements using Mimics Research Version 24.0 (Materialise NV, Leuven, Belgium). We identified individual electrodes using the coronal view (fundus view), and made corresponding measurements using the axial view (B-scans). The opaque platinum electrodes occlude the scanning light, creating a dark shadow on the underlying tissue. This reflection artifact confirmed the exact electrode locations on the B-scans, and we made measurements at the center of the largest shadow obtained by a B-scan. Since the OCTs could not precisely center every electrode in line with a scan pattern, our measurements occurred at the center of the chord that was closest to the full electrode diameter. We measured the retinal thickness as the distance from the inner to outer boundary, without subdividing retinal layers. Figure 5 shows example measurements for two electrodes.

We also obtained electrode impedance and perceptual threshold data for each participant. We used the Argus II Clinician Fitting System (CFS) to measure the impedance of each electrode. We used the Hybrid Threshold program on the CFS to determine perceptual threshold for individual electrodes, as described in prior work^24^. Perceptual threshold is the current amplitude at which the participant sees a percept 50% of the time, based on a Weibull function sigmoid curve fit to “yes”/”no” responses. Electric stimuli were biphasic, cathode-first current pulses with 0.45 ms pulse width and 20 Hz frequency. We used a train of five identical pulses for each trial (250 ms duration). Perceptual threshold collection occurred over a 1–2 day period for each participant.

### Statistical models for perceptual threshold

We used regression analysis to create statistical models for perceptual threshold. An electrode was included in the analysis if it was visible on at least one B-scan and the perceptual threshold was less than 677 µA. This upper threshold cutoff is the safe charge density limit for the Argus II electrodes, corresponding to 1.0 mC/cm^2^/phase^10^. We provide a summary of excluded electrodes in Supplementary Table S1. Explanatory variables were electrode-retina distance (mm), retinal thickness (mm), fibrotic tissue thickness (mm), and electrode impedance (kΩ). We fit individual linear regression models to find the influence of each explanatory variable on perceptual threshold for each participant. We fit a multiple regression model to find the cumulative influence of all explanatory variables on perceptual threshold for each participant. In each case, we calculated the coefficient of determination (R^2^) and *p*-value. We used a significance level (α) of 0.05. We performed statistical analysis using R version 4.3.0 (R Core Team, 2023).

Additionally, we used linear mixed models to determine the influence of explanatory variables on perceptual threshold across participants. We fit a random intercept and slope model for each explanatory variable using the ‘lme4’ package to explore how variation in perceptual threshold depended on fixed effects across individuals and on individual-level random effects. Significance of fixed effect was determined using Satterthwaite's degrees of freedom. Significance of random slope was determined by likelihood ratio test.

### Patient-specific field-cable models for perceptual threshold

Our methodology for creating patient-specific field-cable models was adapted from our prior proof-of-concept study, conducted in a single participant^24^. We created models of the implanted eye for participants with at least one significant explanatory variable identified during regression analysis. As a result, we excluded participants CL01 and CL02 from this portion of the study, and we built five patient-specific field-cable models.

**Figure 6:**
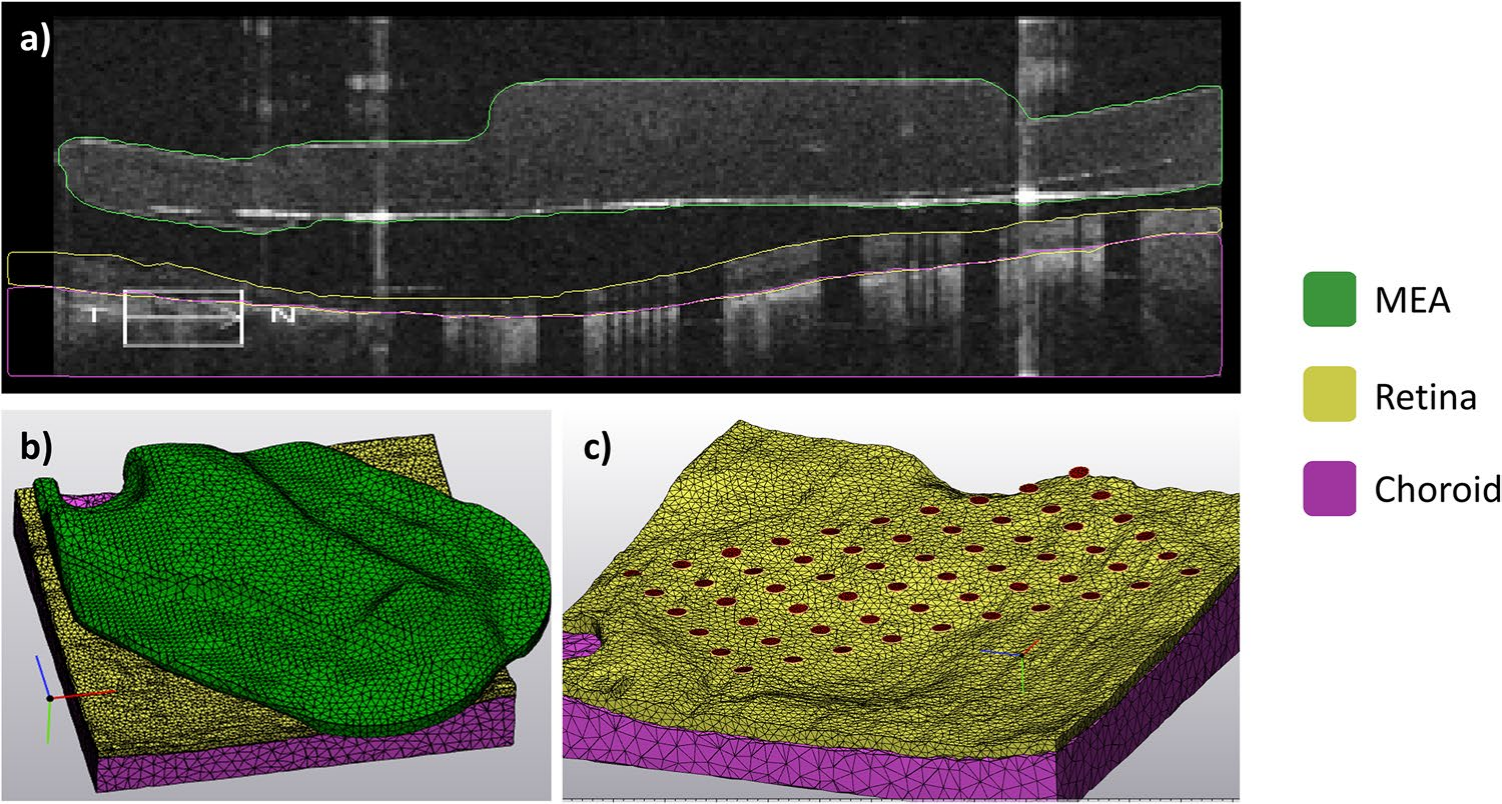
Segmentation and reconstruction. (**a**) Sample segmented B-scan showing the microelectrode array, retina, and choroid. (**b**) Finite element mesh showing a 3D reconstruction of the same three domains. (**c**) Finite element mesh with the MEA substrate hidden to show individual electrode surfaces created by the intersection curve operation.

We segmented OCT scans in Mimics Research Version 24.0 (Materialise NV, Leuven, Belgium) using the multiple slice edit tool. Prior to segmentation, we corrected the jitter in scans obtained with the Zeiss Cirrus system using the image processing toolbox in MATLAB R2021a (MathWorks Inc., Natick, MA, USA). Segmented domains included the microelectrode array (MEA), retina, choroid, and fibrotic tissue (Fig. 6a).

We converted the segmented images into a finite element mesh (FEM) using the 3-Matic Research Version 16.0 (Materialise NV, Leuven, Belgium) (Fig. 6b). Pre-processing included wrapping each domain with a gap closing distance of 25 µm. Sixty circular electrode surfaces were created on the MEA surface by using cylinders to create intersection curves. Cylinders were 200 µm in diameter and spaced at 525 µm pitch and were aligned using the electrode shadow artifacts. The resulting electrode surfaces created by the intersection curve operation are shown in Fig. 6c.

We created a processing pipeline to refine each patient-specific FEM. To limit edge effects, we extruded the edges of the retina and choroid to create a rectangular model with 25 × 17 mm dimensions. We created a vitreous domain that extended 18 mm above the retinal surface. We performed an adaptive re-mesh of all surfaces with a maximum triangle edge length of 0.05 mm for the MEA, and a maximum triangle edge length of 0.1 mm for the retina, choroid, and vitreous. We built a grid-based non-manifold assembly (grid resolution: 0.01 mm) to align the triangle nodes of adjacent surfaces. Finally, we calculated a tetrahedral volume mesh for the entire model. The number of tetrahedral volume elements for each patient-specific FEM was between 3.3 and 4.0 million.

**Figure 7:**
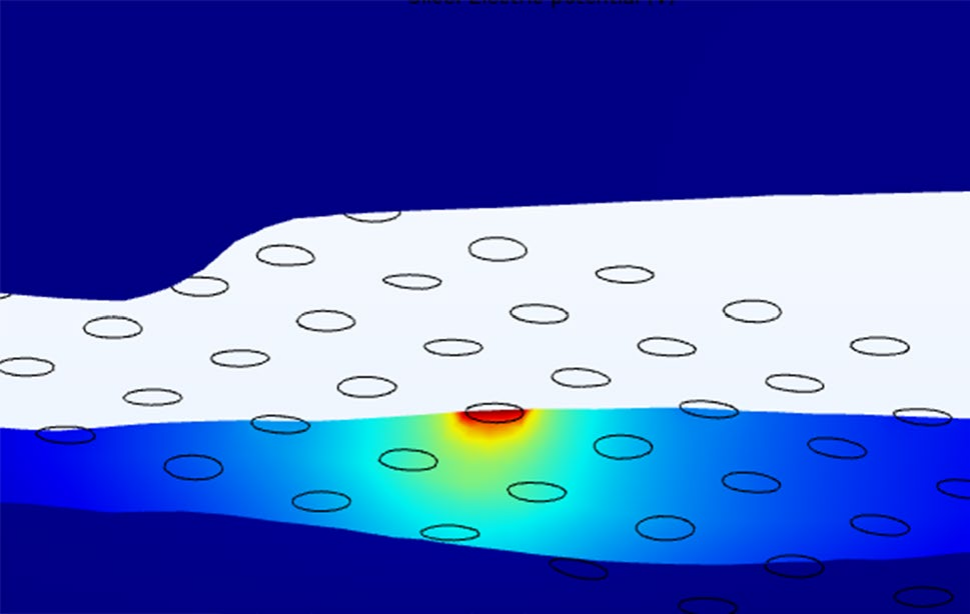
Example of electric potential distribution calculated in the patient-specific FEM for electrode D5, participant CL03.

We conducted finite element analysis in COMSOL Multiphysics Version 5.6 (Stockholm, Sweden) using the AC/DC electric currents (ec) module, following the methods described in our previous work^24^. We represented the active electrode as a surface current terminal (1A) and assigned a floating potential boundary condition to inactive electrodes^31^. We designated the outer boundary of the choroid as the electrical ground (0 V). We assigned a tissue conductivity value to each domain (σ_choroid_ = 0.503 S/m^50^, σ_retina_ = 0.100 S/m^32,51^, σ_vitreous_ = 1.5 S/m^50^, σ_platinum_ = 9.43 × 10^6^ S/m^31^, σ_fibrosis_ = 0.15 S/m^52^). We used a contact impedance condition to model the thin, resistive retinal pigment epithelium membrane at the boundary between the retina and choroid (thickness = 10 µm, σ = 0.001 S/m^32,53^). We modelled the MEA substrate as a perfect insulator (σ = 0 S/m). We used a quasi-static solver to calculate the electric potential distribution throughout the finite element mesh by solving Laplace’s equation (Fig. 7). Solving the FEM for a single active electrode takes between three to five minutes on a desktop computer with 32 GB RAM.

**Figure 8:**
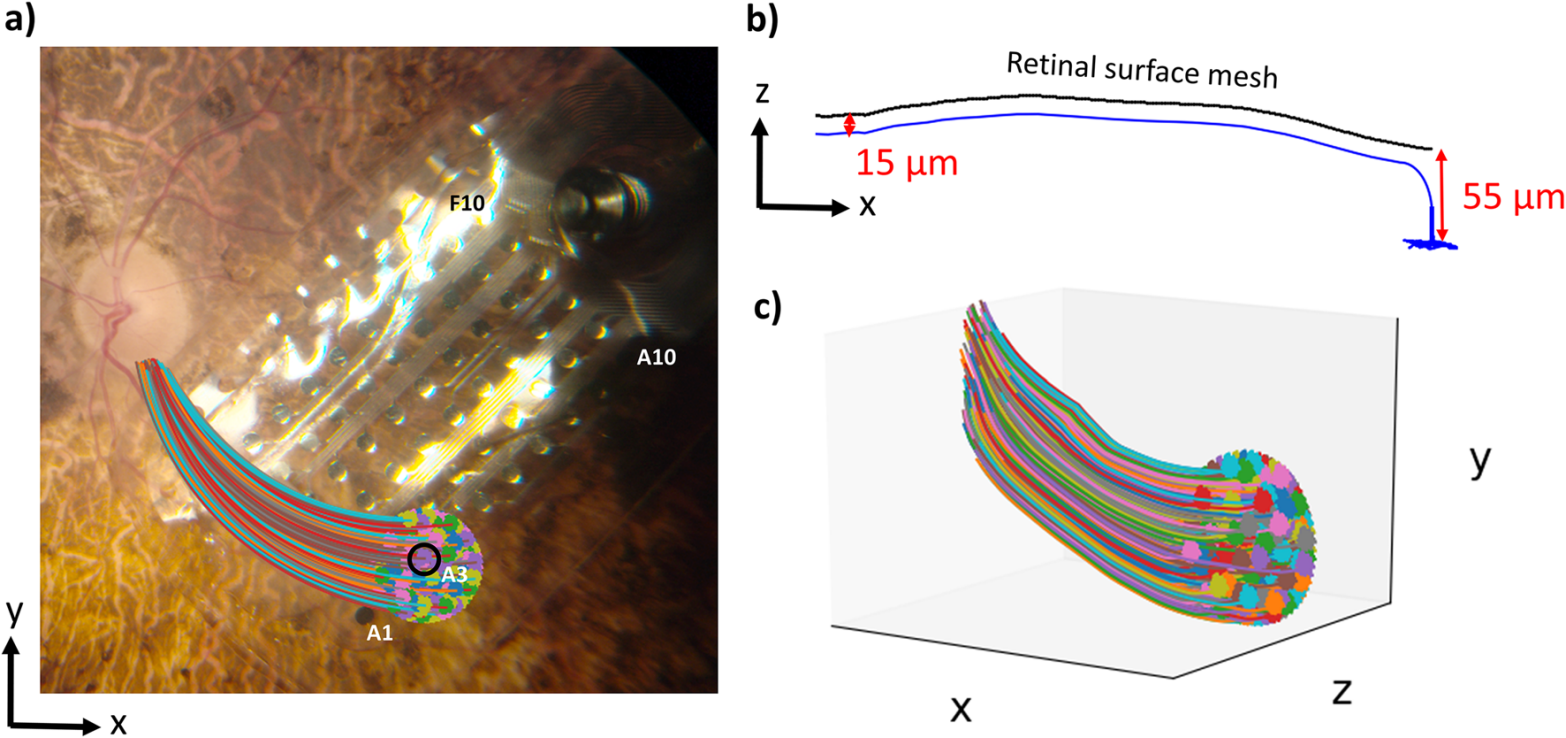
Neuron placement paradigm (**a**) The x–y coordinates of a target retinal ganglion cell population beneath electrode A3 for participant UM01. Somas were distributed in a 700-µm circular region beneath the electrode, and axon x–y coordinates were calculated using equations defined by Jansonius et al.^55^ (**b**) The z-coordinates for a single axon, calculated to keep the RGC within the retinal domain of the finite element mesh. (**c**) The 3-D coordinates of the entire target RGC population (250 neurons) for electrode A3.

To predict the neural retina’s response to stimulation, we populated the retinal domain of the FEM with multi-compartment cable models of retinal ganglion cells. For each electrode in our FEM, we uniformly distributed 250 somas within a 700 µm radius beneath the electrode using Lloyd’s algorithm^54^. We calculated axon trajectories using the equations defined by Jansonius et al.^55^ (Fig. 8a). We positioned each soma 55 µm below the retinal surface mesh and positioned each axon 15 µm beneath the retinal surface mesh (Fig. 8b). Figure 8c shows the coordinates of an example target RGC population for one electrode.

The cable equations for RGC membrane dynamics are described in detail in our prior publication, and our biophysical model is open-source on GitHub^45^. Briefly, we determined the extracellular potential at the center of each neuron compartment from the FEM solution. We scaled these extracellular potentials by the stimulus waveform, and applied them to the neurons, integrating over time to calculate the membrane voltage response. To match clinical experiments, electric stimuli were biphasic, cathode-first current pulses with 0.45 ms pulse width. We found the action potential threshold for each individual neuron using a bisection algorithm with a convergence of 0.1 µA.

We found the absolute minimum action potential threshold for any neuron in each electrode’s target RGC population. We used the same electrode inclusion criteria as described for the statistical model. By using a uniform distribution of neurons, our patient-specific field-cable models did not capture the influence of retinal thickness on perceptual threshold. To remedy this, we removed neurons in regions where the retina had degenerated to a thickness less than 100 µm^56^. We re-computed the absolute minimum action potential threshold in each electrode’s target RGC population. We compared the model-predicted thresholds to each participant’s perceptual threshold using linear regression.

### Phosphene predictions

We compared the patient-specific field-cable model predictions of retinal activation to a prior study analyzing the effect of increasing stimulation amplitude on phosphene size^8^. Nanduri et al. collected phosphene drawings in response to single-electrode stimulation from nine epiretinal disc electrodes^8^. In concordance with our study, electric stimuli were biphasic, charge-balanced, 0.45 ms/phase, cathode-first pulse trains that were 500 ms in duration^8^. Stimulus amplitude was modulated between 1.2 × and 6 × threshold, while holding frequency constant at 20 Hz^8^. The study found that increasing pulse amplitude caused an increase in phosphene size at an average rate of 1.17 deg^2^/X threshold (range of slopes: 0.73–1.92)^8^. 

To replicate this study in our patient-specific field-cable models, we chose a single electrode from each model. We calculated the retinal area activated as stimulus amplitude was modulated between 1.2 × and 6 × threshold. We calculated retinal area (mm^2^) using the “shapely.MultiPoint” tool in Python to fit a convex hull around activated cell bodies (Supplementary Figure S1). Retinal area was converted to degrees of visual angle using a conversion factor of 288 µm per degree^57^.

### Supplementary Information

Supplementary Information.

## Data Availability

The datasets generated during and/or analyzed during the current study are not publicly available out of concern for patient privacy, as data from medical records is considered sensitive, but de-identified datasets are available from the corresponding author on reasonable request.

## Code availability

The computer code used to model the extracellular stimulation of retinal ganglion cells using cable equations is available at: https://github.com/Kathleen-Kish/Retinal_Ganglion_Cell.
