# Supplementary material for: Patient-specific computational models of retinal prostheses
Source: Sci Rep. 2023 Dec 14;13:22271. doi: 10.1038/s41598-023-49580-6 (PMC10721907; doi:10.1038/s41598-023-49580-6)
Supplement: Supplementary file 1 — Supplementary Information. [file 41598_2023_49580_MOESM1_ESM.docx]

**Supplementary Table S1.** Electrode exclusion summary

| **Participant ID** | **Implant eye** | **Number of electrodes included in study / Total number of electrodes measured** | **Excluded electrodes** |
| --- | --- | --- | --- |
| UM01 | Left | 27 / 30 | Not visible on OCT (3) |
| UM02 | Left | 26 / 30 | Not visible on OCT (4) |
| CL01 | Right | 56 / 60 | High impedance (1)  Not visible on OCT (3) |
| CL02 | Right | 45 / 60 | High impedance (1)  Threshold >677 µA (12)  Not visible on OCT (2) |
| CL03 | Right | 54 / 60 | High impedance (2)  Not visible on OCT (4) |
| CL04 | Right | 35 / 60 | High impedance (7)  Threshold >677 µA (11)  Not visible on OCT (7) |
| CL05 | Left | 47 / 60 | High impedance (5)  Not visible on OCT (8) |


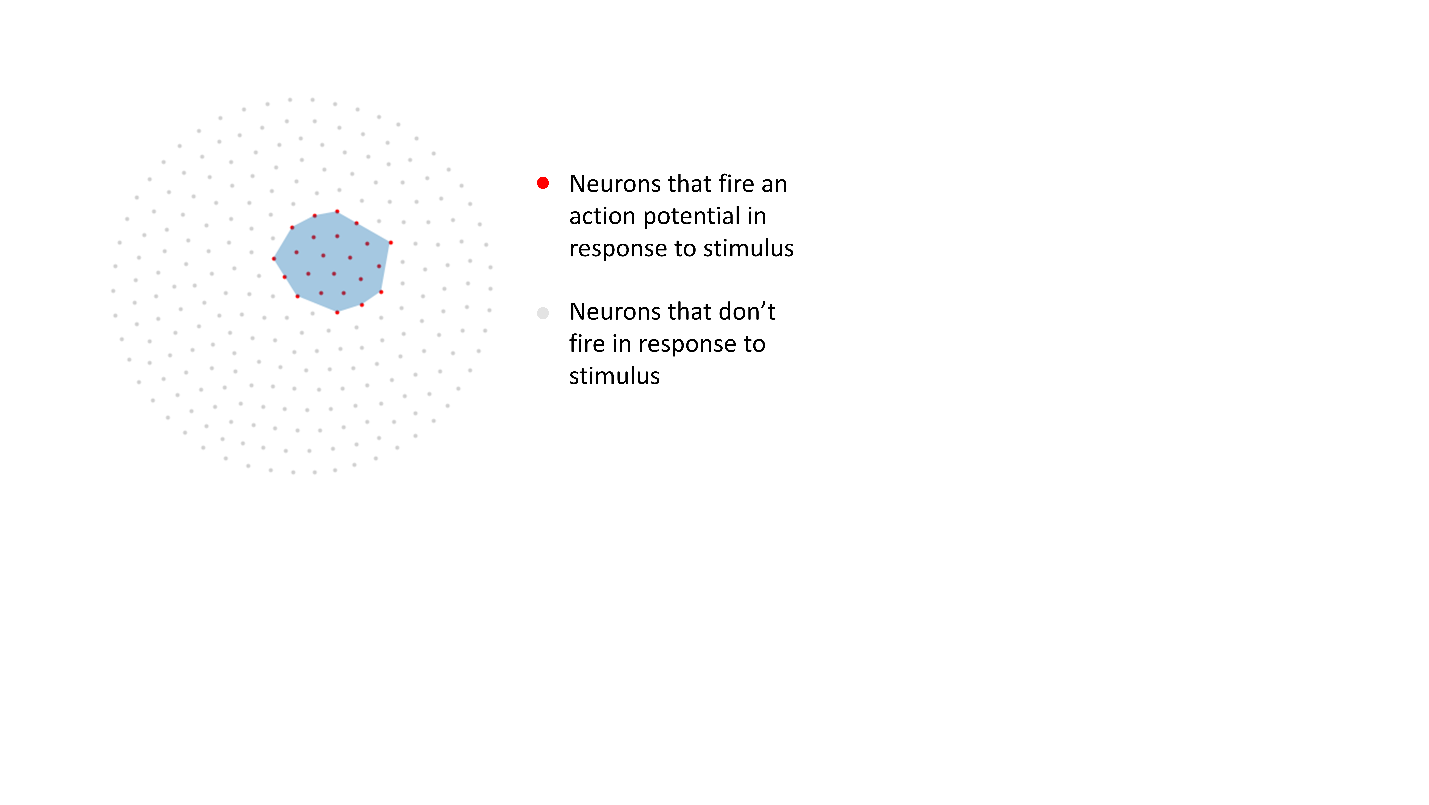


**Supplementary Figure S1.** Method for calculating retinal area activated (mm^2^)
